# Full-Genome Hepatitis B Virus Genotyping: A Juxtaposition of Next-Generation and Clone-Based Sequencing Approaches—Comparing Genotyping Methods of Hepatitis B Virus

**DOI:** 10.3390/v18010112

**Published:** 2026-01-15

**Authors:** Li-Ping Hu, Qin-Yan Chen, Mei-Lin Huang, Wen-Jia Zhang, Xiao-Qian Huang, Xian-Feng Yi, Hui-Hua Jia

**Affiliations:** 1 Guangxi Zhuang Autonomous Region Center for Disease Prevention and Control, Guangxi Key Laboratory for the Prevention and Control of Viral Hepatitis, Nanning 530028, China; wfhlp2007@163.com (L.-P.H.); chenqingyan060@sina.com (Q.-Y.C.); ml-huang@outlook.com (M.-L.H.); zwenjia771@163.com (W.-J.Z.); gdzhhxq@163.com (X.-Q.H.); 2 The Animal Husbandry Research Institute of Guangxi Zhuang Autonomous Region, Nanning 530010, China; 3 Drug and Food Vocational College, Guangxi Vocational University of Agriculture, Nanning 530007, China

**Keywords:** hepatitis B virus, genotyping, next-generation sequencing (NGS), clone-based sequencing (CBS), recombinant

## Abstract

**Background:** The enhanced sensitivity of next-generation sequencing (NGS) for assessing hepatitis B virus (HBV) quasispecies heterogeneity over clone-based sequencing (CBS) is well documented. However, its comparative reliability for genotype determination remains an open question. **Objective:** This study aimed to directly compare the performance of NGS and CBS for genotyping HBV using the entire viral genome. **Methods:** We selected five challenging clinical samples that previously could not be subgenotyped or showed conflicting results when using direct sequencing of the S open reading frame (ORF). The full HBV genome from these subjects was amplified and then analyzed in parallel by both NGS and CBS. Phylogenetic analysis was subsequently used to assign genotypes. **Results:** Both methods identified a range of genotypes, including B, C, and I, as well as aberrant and recombinant forms. For three of the five subjects, genotyping results were identical between the two platforms. In the remaining two cases, however, CBS revealed greater complexity, identifying additional subgenotypes and recombinant/aberrant strains not detected by NGS. Notably, for three individuals, the genotypes determined by both modern methods contradicted earlier results from 2011 based on direct S ORF sequencing. Furthermore, the specific mutations detected were incongruent between the platforms, with CBS identifying a higher number of variants than NGS. **Conclusions:** Our findings indicate that genotyping results from NGS and CBS can be discordant. Contrary to expectations, CBS may uncover more genetic diversity, including a greater number of subgenotypes and mutations, than NGS in certain contexts. The study also confirms that genotyping based solely on direct sequencing of the S ORF can be unreliable and lead to misclassification.

## 1. Introduction

Hepatitis B virus (HBV) has a partially double-stranded, circular DNA genome of approximately 3200 base pairs, including four partly or completely overlapping open reading frames (ORFs): preC/C, P, PreS1/S2/S, and X [1]. The encoded reverse transcriptase lacks proofreading activity, resulting in a high genetic heterogeneity, with various genotypes and subgenotypes and the development of viral quasispecies in individual infections [2].

The term quasispecies denotes a swarm of closely genetically related variants, where the nucleotide variation is less than between genotypes and subgenotypes [3]. Nine distinct genotypes of the hepatitis B virus are currently recognized, a classification stemming from comprehensive phylogenetic and evolutionary investigations of entire genome sequences. Each of these genotypes exhibits a nucleotide variation of over 7.5% when compared to the others [4,5]. A more refined categorization has been established for genotypes A–D, F, and I, which are now divided into no fewer than 55 subgenotypes. This subdivision is based on an internal nucleotide variation of roughly 4–8% at the complete genome level and is validated by significant bootstrap statistical support [1,6,7,8].

Hepatitis B virus genotypes are not uniformly distributed globally, showing clear geographical segregation. A prime example is genotype A, which is predominantly found in populations across Africa, Europe, India, and the Americas. Meanwhile, the Asia-Pacific area is characterized by the common occurrence of both genotypes B and C [9]. It has been reported that infections with different genotypes have various clinical implications. While the effectiveness of direct antiviral medications for HBV shows little consistent variation among genotypes, a clear disparity exists for interferon-based therapy. Specifically, patients with genotypes A and B tend to respond more successfully to interferon treatments than those carrying the C and D genotypes [10]. Infection with hepatitis B virus genotypes C, D, and F confers a more substantial lifetime risk for progression to cirrhosis and hepatocellular carcinoma, in contrast to infections involving genotypes A and B [11].

For the determination of HBV genotypes, a multitude of techniques have been put into practice over time. These range from restriction fragment mass polymorphism (RFMP) and PCR-invader assays to more common procedures like real-time PCR and the direct sequencing of PCR products. Hybridization-based tools, including INNO-LiPA strips and reverse dot blot assays, also serve this function. Nevertheless, the benchmark method for accurately assigning an HBV genotype is the sequencing of the full viral genome, followed by a detailed phylogenetic comparison [4,12]. A more cost-effective approach is to sequence an individual ORF (e.g., the surface ORF) rather than the complete genome [13].

A combination of molecular cloning of PCR products and subsequent Sanger dideoxy sequencing represents a viable technique for genotyping in complex cases involving mixed HBV infections [14]. Clone-based sequencing (CBS), often considered the gold standard, involves isolating a single DNA molecule by inserting it into a bacterial plasmid and growing it into a clonal population. This process allows for the determination of a consensus sequence from thousands of identical copies, yielding long, high-fidelity reads (800–1000 bases) and the unique ability to resolve haplotype phases. However, CBS is low-throughput, time-consuming, and costly for large-scale projects. In contrast, Next-generation sequencing (NGS) employs a massively parallel strategy. DNA is fragmented into short pieces, and all fragments are sequenced simultaneously in a single run. This approach generates an enormous volume of data at a low cost per base, making it ideal for profiling entire genomes or transcriptomes. The primary trade-offs are shorter read lengths (50–300 bases), which can complicate assembly in complex genomic regions, and the loss of direct haplotype information [15,16]. The ability to detect the complex diversity within HBV viral populations is significantly enhanced by using next-generation sequencing, which the literature has reported to be more sensitive than clone-based sequencing techniques [17]. However, it is unclear which approach is better for identifying genotypes based on complete genome sequences. We found in our previous study in Guangxi, China, that some isolates could not be subgenotyped or yielded different genotypes using S gene sequences, with direct sequencing of different PCR products [18]. Genotypes in this region are complex; genotypes C, B, and I (a recombinant) are the most common [18,19,20]. In order to determine the superior methodology, this study will ascertain the genotype and subgenotype of each sample through the parallel application of next-generation sequencing and conventional clone-based sequencing.

## 2. Materials and Methods

### 2.1. Study Subjects and Sample Collection

The study subjects were selected from a study cohort, which has been described previously [21]. We found that isolates from five study subjects could not be subgenotyped or yielded different genotypes using S gene sequences with direct sequencing of different PCR products [18]. Serum samples collected in 2011 from the five study subjects were included in the analysis. For comparison, we also collected serum samples from these study subjects in 2020. This study was conducted in strict adherence to the ethical tenets of the 1975 Declaration of Helsinki and received formal approval from the Guangxi Institutional Review Board. Prior to enrollment, every participant provided their written informed consent. A key exclusion criterion for all subjects was the presence of co-infection with either hepatitis C virus (HCV) or human immunodeficiency virus type 1 (HIV-1).

### 2.2. Serological Testing

Serum samples were evaluated for both viral serological markers and a key liver enzyme. Specifically, enzyme immunoassays (EIA; Beijing Wantai Biopham Company Limited, Beijing, China) were utilized to detect HBsAg and HBeAg/anti-HBe, while alanine aminotransferase (ALT) activity was quantified with a Reitman kit (DiaSys Diagnostic Systems, Shanghai, China).

### 2.3. Measurement of Serum Viral Loads

Real-time polymerase chain reaction (PCR) was used to quantify the concentration of HBV DNA in serum samples. This quantification was carried out on an ABI Prism 7500 instrument (Applied Biosystems, Foster City, CA, USA) using a commercial kit from Sansure Biotech Inc. (Changsha, China), which provided a linear detection range of 1 × 10^2^ to 5 × 10^9^ IU/mL.

### 2.4. Nested Polymerase Chain Reaction (PCR) for HBV DNA

HBV genomic DNA was extracted from 200 μL of serum samples using QIAamp DNA Mini kits (QIAGEN GmbH, Hilden, Germany) and eluted in 50 μL of distilled water. The entire HBV genome was amplified using nested PCR. The amplification protocol and primers P1 (nt 1821–1841, 5′-CCGGAAAGCTTGAGCTCTTCTTTTTCACCTCTGCCTAATCA-3′) and P2 (nt 1823–1806, 5′-CCGGAAAGCTTGAGCTCTTCAAAAAGTTGCATGGTGCTGG-3′) have been described previously [22]. The initial amplification was conducted using a VeritiPro Thermal Cycler (Thermo Fisher Scientific, Waltham, MA, USA) for a total of 40 cycles. The thermal profile included denaturation at 94 °C for 40 s, annealing at 60 °C for 1.5 min, and an elongation phase at 68 °C for 3 min. Notably, the elongation time was progressively lengthened by two minutes following each block of ten cycles. Subsequently, a second-round PCR was initiated using 5 μL of the primary amplicon in a 50 μL reaction volume. This nested reaction featured primers MDN5R (nt 1774-1794, 5′-ATTTATGCCTACAGCCTCCT-3′) and BCPF (nt 1854–1875, 5′-ATGTCCTACTGTTCAAGCCTCC-3′). Its protocol began with a 5 min hot start and then proceeded for 30 cycles of 94 °C for 30 s, 50 °C for 30 s, and 72 °C for 4 min. For samples characterized by low viral titers, a preliminary rolling circle amplification step was implemented before the nested PCR procedure [23], which exhibits less amplification bias and greater yield, product length, and fidelity than PCR [24,25]. Confirmation of the second-round PCR output was achieved by analyzing the products via electrophoresis with a gel composed of 1% agarose.

In order to obtain sequences (60 nt in total) between the positions of primer P1 and BCPF and P2 and MDN5, two second round PCRs were carried out on 5 μL of the first round’s products above in a 50 μL reaction using primers P1 and MDC1 (nt 2304–2324, 5′-TTGATAAGATAGGGGCATTTG-3′) and P2 and XSEQ3(nt 1653–1672, 5′-CATAAGAGGACTCTTGGACT-3′), respectively. The PCR program is a 5 min hot start followed by 30 cycles of 94 °C for 30 s, 50 °C for 30 s, and 72 °C for 30 s.

### 2.5. Clone-Based Sequencing

**Figure 1:**
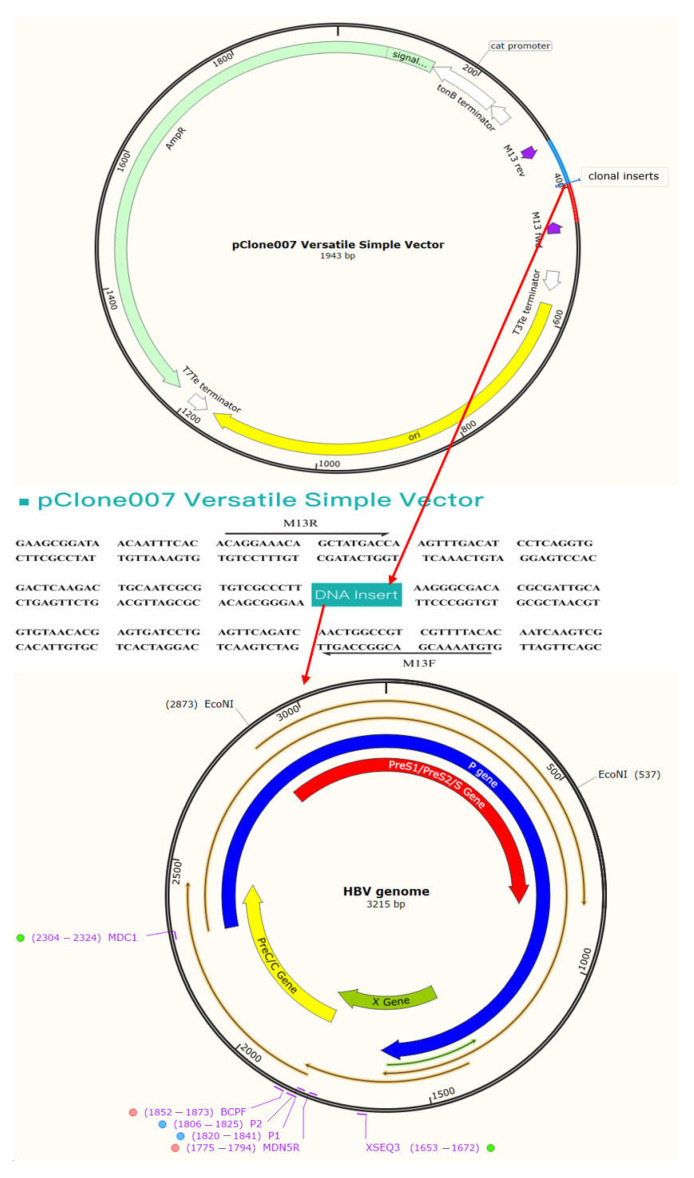
The cloning vector P clone 007T and the HBV clone insertion sites.

Amplicons from the second round were confirmed by agarose gel electrophoresis and cloned into the vector P clone 007T (The Beijing Qingke Biotech Co., Ltd., Beijing, China) according to the manufacturer’s instructions and subsequently transformed into competent *Escherichia coli* (The Beijing Qingke Biotech Co., Ltd., China) (Figure 1). Following plasmid DNA extraction with a SK1191 UNIQ-10 kit (The Beijing Qingke Biotech Co., Ltd., China), the purified samples were sequenced. The analysis was completed by The Beijing Qingke Biotech Co., Ltd., where the reaction was performed using the BigDye Terminator V3.1 Cycle Sequencing kit from Applied Biosystems (Foster City, CA, USA).

For the direct sequencing of small PCR fragments, a 2 µL aliquot of purified amplicon DNA served as the template. The procedure, carried out at The Beijing Qingke Biotech Co., Ltd. (China), employed primers MDC1 and XSEQ3 along with the BigDye Terminator V3.1 Cycle Sequencing kit (Applied Biosystems, Foster City, CA, USA), strictly following the kit manufacturer’s instructions.

Sequences were determined for both strands to derive robust data for comparison with the full-length sequences of the various genotypes.

Forward primers:

W803-C01 (nt 43–61, 5′-GGGGCCTGTATTTTCCTGCT-3′),

W798-A02 (nt 254–273, 5′-TGTCAACAATTTGTGGGCCC-3′),

W807-F05 (nt 505–524, 5′-ATTCCTATGGGAGTGGGCCT-3′),

W803-A01 (nt 810–829, 5′-ACCAATCGGCAGTCAGGAAG-3′),

W811-C09 (nt 1252–1271, 5′-GCTCCTCTGCCGATCCATAC-3′).

W798-B02 (nt 1633–1652, 5′-TGTGAACAATTTGTGGGCCC-3′),

W798-E01 (nt 2472–2491, 5′-GTGGGAAACTTTACCGGGCT-3′),

W803-B01 (nt 3061–3080, 5′-GGAGGTCTTTTGGGGTGGAG-3′),

Reverse primers:

W807-A01 (nt 60–41, 5′-GCAGGAAAATACAGGCCCCT-3′),

W807-B01 (nt 353–334, 5′-GGACAGGAGGTTGGTGAGTG-3′),

W807-C01 (nt 893–874, 5′-CCCCAATCCTCGCGAAGATT-3′).

W798-C02 (nt 1237–1218, 5′-CCACAAAGGTTCCACGCATG-3′),

W803-D01 (nt 1543–1524, 5′-GAGGCCCACTCCCATAGGTA-3′),

W803-B03 (nt 2325–2306, 5′-AGGCCCACTCCCATAGGAAT-3′),

W798-F01 (nt 2640–2621, 5′-GTATGGATCGGCAGAGGAGC-3′),

### 2.6. Workflow for Next-Generation Sequencing Analysis

For high-throughput sequencing, amplicons from the secondary PCR were processed at Delivectory Biosciences Inc. (Beijing, China). Following an initial purification step with Agencourt AMPure XP beads (Beckman Coulter, Shanghai, China) and quantification using Qubit dsDNA HS assay kits (Invitrogen, Carlsbad, CA, USA), DNA libraries were constructed. The Celero EZ DNA-Seq Library Preparation Kit (Tecan Genomics, Shanghai, China) was utilized for this purpose before the samples were sequenced on an Illumina Noveseq platform, per the manufacturer’s instructions. The system’s control software analyzed the raw optical data, which was ultimately transformed into paired-end reads with sequences of 2 × 150 base pairs.

### 2.7. NGS Data Preprocessing and Sample Genotyping

Quality control and preprocessing of each sample’s raw NGS short reads were performed by fastp v0.20.1 [26]. The raw sequence reads underwent several data processing steps, beginning with the trimming of adapters and the excision of 15 bases from the 5′ end. A filtering process was then applied to remove reads with a length under 50 nucleotides or an average quality score below 30. Using the bowtie2 alignment tool (v2.3.4.1), the remaining high-quality reads from each sample were subsequently mapped to the reference sequence X02763 [27]. First, the Samtools v1.7 utility [28] was applied to sort the alignments and filter out any duplicate reads. The final consensus sequence was subsequently generated from this processed dataset with the CliqueSNV v1.5.3 tool [29].

### 2.8. Haplotype Inference and Quasispecies Diversity Assessment

To assess diversity and construct haplotypes, the filtered NGS reads for each sample were first re-mapped against appropriate genotype-specific references. This alignment was performed with bowtie2’s very-sensitive-local mode [27], followed by the removal of duplicates using Sambamba [30]. Haplotype structures were then inferred from the processed SAM alignment files employing the CliqueSNV software (v1.5.3) with all settings at their default values [29]. A threshold of 1% minimum abundance was set for a haplotype to be included in subsequent investigations, and each was treated as a unique HBV variant’s genome. Ultimately, the heterogeneity of the viral quasispecies was quantified through its genetic complexity, a metric based on the number of distinct sequences identified.

#### 2.8.1. HBV Genotype Determination

Phylogenetic reconstruction, based on both the complete genome and the preS/S gene regions, was the method used for HBV genotype classification. Our sequences were first aligned to 46 GenBank reference sequences of known genotypes with the Clustal W tool, and the result was visually inspected using BioEdit [31] (reference details are shown in Table 1). Subsequently, maximum likelihood trees were built with the MEGA_12.0.11 software [32] employing the GTR+I+G substitution model. The reliability of the clustering was established by performing an interior branch test of 1000 replicates, where a support threshold of 75% for internal nodes was considered significant.

#### 2.8.2. Detection of Recombination

To investigate potential genetic exchange, a recombination analysis was carried out with the Simplot program (V.3.5.1) using its boot scanning function, consistent with our earlier study. The complete HBV sequence was scanned against consensus sequences representing genotypes A–I. During the bootscan, the query sequence’s phylogenetic position was evaluated relative to reference parental strains (FR714490, AB074047) and two outgroups (AY226578, AB486012). A shift in the supported phylogenetic clustering along the genome was interpreted as evidence of a recombination event. This process was executed with a window length of 400 base pairs, a 20 base pair step size, and a bootstrap value of 1000 replicates.

## 3. Results

### 3.1. General Information

The five study subjects were two males and three females with an average age of 57.2 ± 10.5 years (range from 31 to 66). All were negative for HBeAg and had ALT levels below 40 U/L. The median of viral load was 4.06 × 10^2^ IU/mL (IQR: 88 IU/mL~4.59 × 10^2^ IU/mL) (Table 2). Although complete genome sequences were obtained from all study subjects, sequences were not derived from the same individual at two time points (2011 and 2020). The major problems were that some samples were of low volume and others had viral loads below the detection limit of PCR.

The average number of clones selected per sample was 29, with a total of 145 clones. In next-generation sequencing, 0.58G raw reads were generated. The average number of quasispecies per sample obtained after filtration and error correction was 9.6, and the total number was 48.

#### 3.1.1. Comparison of CBS and NGS for Genotype/Subgenotype Analysis Based on Complete Genome Sequences

**Figure 2:**
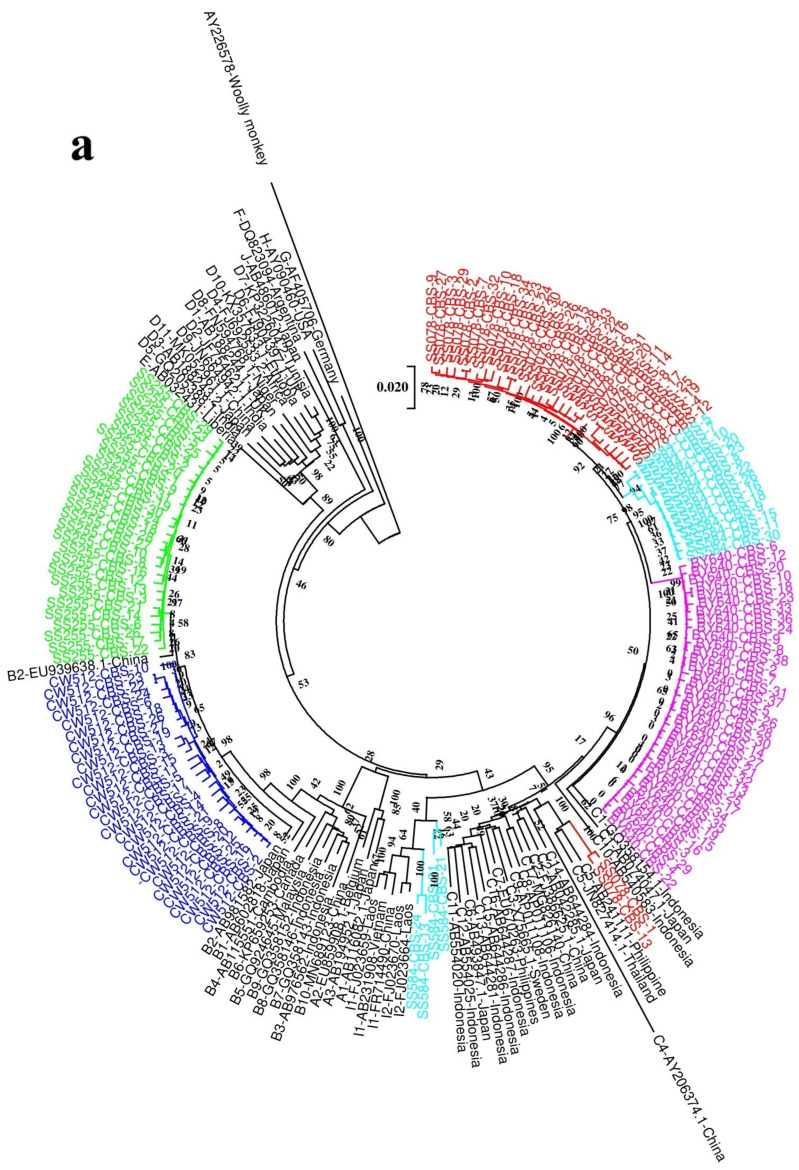

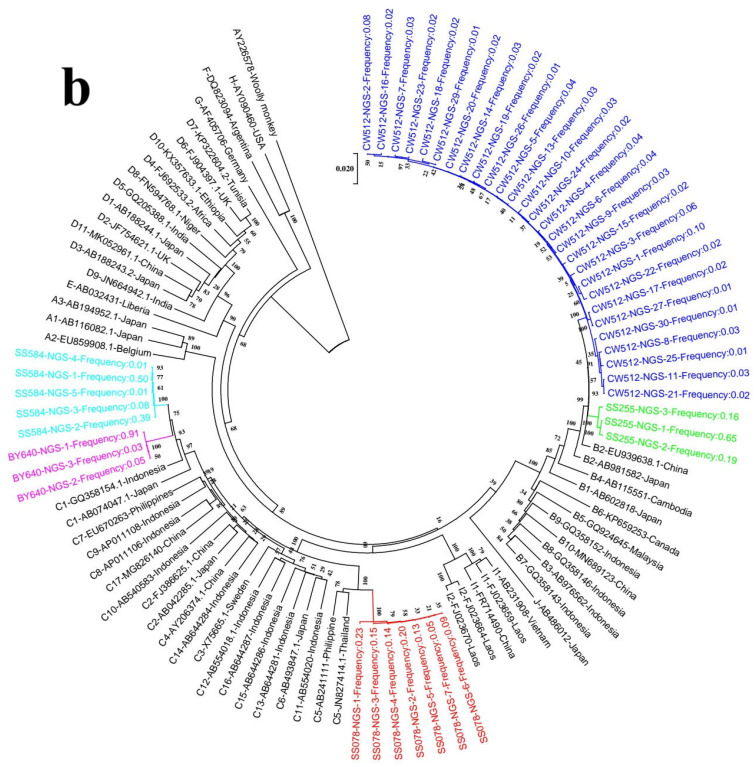
Phylogenetic analysis of CBS (**a**) and NGS (**b**) complete sequences. Maximum likelihood tree was constructed using the complete sequences of the viruses under the GTR+G+I substitution model with the program Mega V7.0. The branch lengths represent the number of substitutions per site. The reliability of clusters was evaluated using the interior branch test with 1000 replicates, and internal nodes with over 75% support were considered reliable. To facilitate interpretation, samples are color-coded by their designated genotype (SS078, red; SS255, green; SS584, blue; BY640, purple; CW512, deep azure).

**Figure 3:**
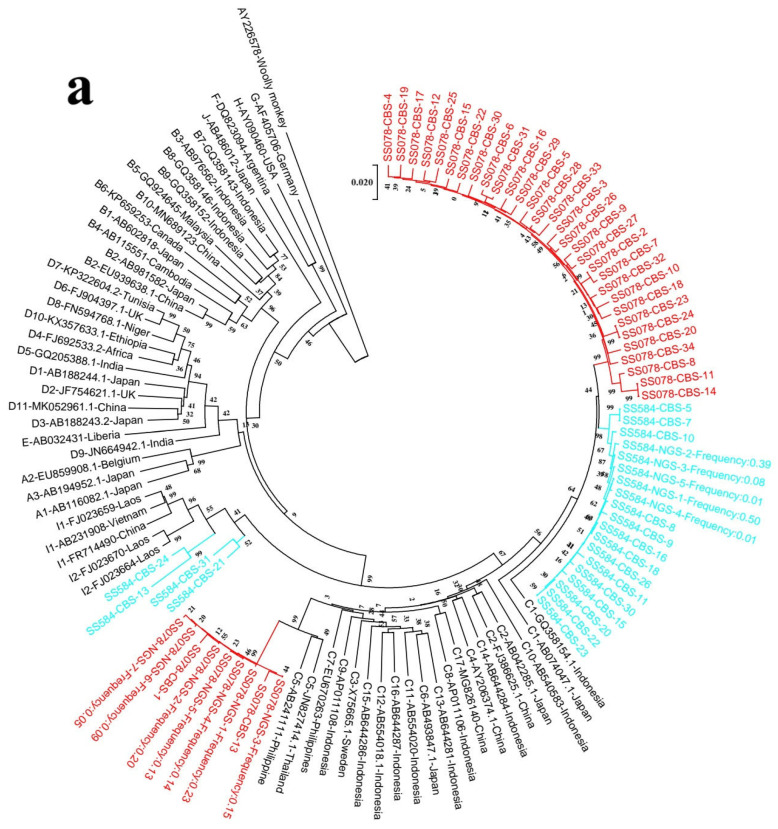

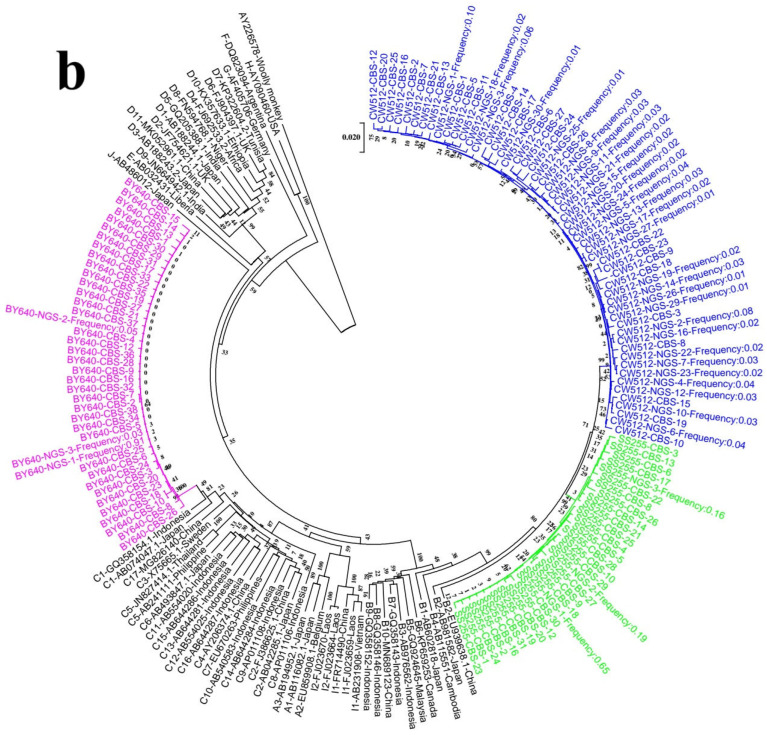
Phylogenetic analysis of discordant samples. (**a**) Concordant samples (**b**) based on CBS and NGS complete sequences. Maximum likelihood tree was constructed using the complete sequences of the viruses under the GTR+G+I substitution model with the program Mega V7.0. The branch lengths represent the number of substitutions per site. The reliability of clusters was evaluated using the interior branch test with 1000 replicates, and internal nodes with over 75% support were considered reliable. To facilitate interpretation, samples are color-coded by their designated genotype (SS078, red; SS255, green; SS584, blue; BY640, purple; CW512, deep azure).

Two phylogenetic trees were constructed on the basis of the complete genome sequences obtained from NGS and CBS (Figure 2 and Figure 3). Genotypes B, C, and I were found with NGS and CBS. In addition, we found a recombinant or an aberrant form of I by CBS. The genetic distances between these strains and all subgenotypes of I exceeded 4%. It is suggested these strains may be recombinant or an aberrant form of I. There are three subjects whose genotype/subgenotype identified with NGS are the same as that identified with CBS. Two subjects have more subgenotypes identified with CBS compared to NGS (Table 3).

All quasispecies of subject SS078 obtained from NGS were typed as subgenotype C5, while only two and almost all of the rest quasispecies obtained from CBS were subgenotype C5 and C1, respectively. In subject SS584, all quasispecies obtained from NGS and almost all quasispecies obtained from CBS were subgenotype C1. One more recombinant and aberrant strain was identified with CBS in that subject. Clearly, genotyping results with sequences obtained from NGS are not consistent with those yielded from CBS. CBS may identify more subgenotypes than NGS.

#### 3.1.2. Comparison of Genotype/Subgenotypes Based on the S ORF Sequences Derived Using NGS, CBS, and Direct Sequencing

**Figure 4:**
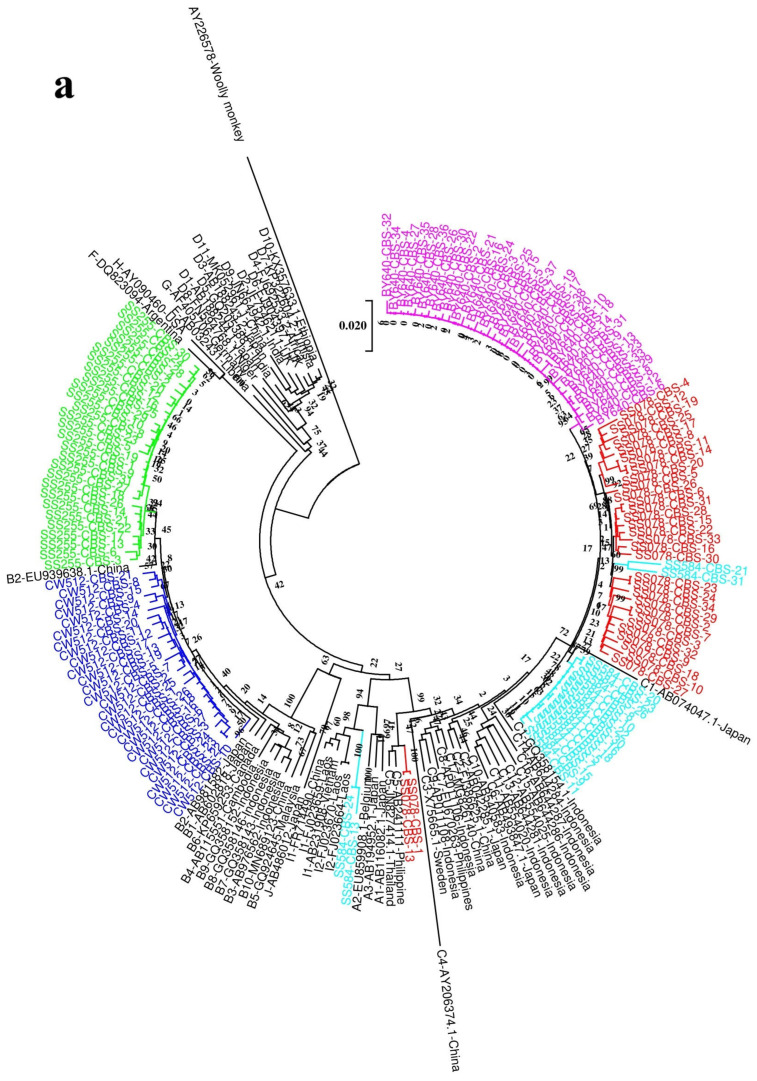

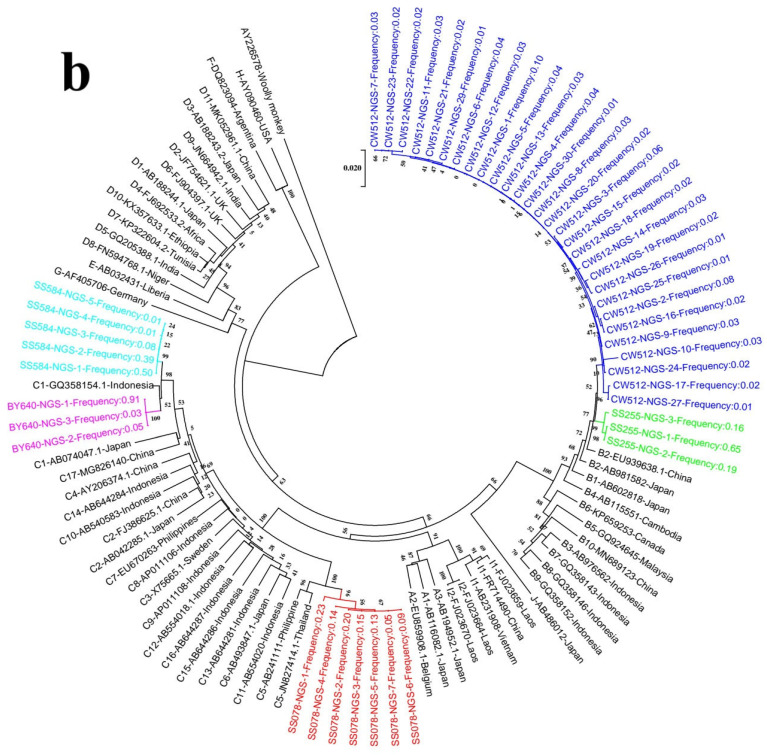
Phylogenetic analysis of CBS (**a**) and NGS (**b**) preS/S sequences. Maximum likelihood tree was constructed using the preS/S sequences of the viruses under the GTR+G+I substitution model with the program Mega V7.0. The branch lengths represent the number of substitutions per site. The reliability of clusters was evaluated using the interior branch test with 1000 replicates, and internal nodes with over 75% support were considered reliable. To facilitate interpretation, samples are color-coded by their designated genotype (SS078, red; SS255, green; SS584, blue; BY640, purple; CW512, deep azure).

In order to clarify the accuracy of genotyping based on the S ORF with direct sequencing, two phylogenetic trees were constructed on the basis of PreS/S sequences obtained from the full-length NGS and CBS sequence (Figure 4). Genotypes/subgenotypes identified from the complete genome and S ORF sequences, obtained by NGS, were the same in all subjects. The same could be seen using CBS in all subjects, except for subject SS584. Complete genome sequencing revealed that this subject was infected with one more aberrant (Table 3).

The genotype/subgenotypes identified in one subject by direct sequencing were the same as those identified from sequences obtained by both NGS and CBS. However, genotype/subgenotypes identified by direct sequencing in four subjects could not be found using NGS or CBS. Subject SS078 had genotype B by direct sequencing, but his genotype was genotype C by both NGS and CBS. Subject BY640 had genotype G or I by direct sequencing, while the genotype was C according to both NGS and CBS. The genotype of subject CW512 could not be genotyped by direct sequencing but was B by NGS and CBS, respectively. Subject SS584 had genotype C or B by direct sequencing, but his genotype was genotype C, an aberrant by both NGS and CBS.

These findings suggest that genotyping based on the S ORF may not be reliable in regions with complex genotypes (Table 3).

#### 3.1.3. Comparison of NGS and CBS for Detection of Mutations in the Complete HBV Genome

All of the mutations found in this study were point mutations. In NGS, mutations were detected by the Samtools mpileup algorithm and another in-house script. Detailed information on variants with read depths greater than 1000 and mutation rates higher than 1.0% is shown in Table 4. In CBS, mutations were determined by comparison with the database (Geno2pheno hbv, https://hbv.geno2pheno.org/ accessed on 24 December 2024). The number of mutations detected by both NGS and CBS was 19. The number of mutations detected by one method was only 13 for CBS and 1 for NGS. Mutations found by NGS were not consistent with those found by CBS. More mutations were found by CBS than by NGS.

Except for subject SS584, all have the PreC nt 1896 (G → A) point mutation according to both NGS and CBS. Except for subject CW512, all have BCP nt 1762 (A → T) and 1764 (G → A) double mutations by NGS or CBS. Subjects SS078 and SS584 have escape mutations by CBS, while none of the sequences obtained from NGS have the same mutation.

The statistical results showed that CBS had significantly better detection ability than NGS (*p* = 0.002) in the C zone (such as L100I, P130T), indicating that CBS was more sensitive to low-frequency mutations. There was no significant difference between NGS and CBS in detecting point mutations in S, RT, X, PreC, and BCP regions (*p* > 0.05).

#### 3.1.4. Analysis of Genetic Recombination and Putative Breakpoint Locations

**Figure 5:**
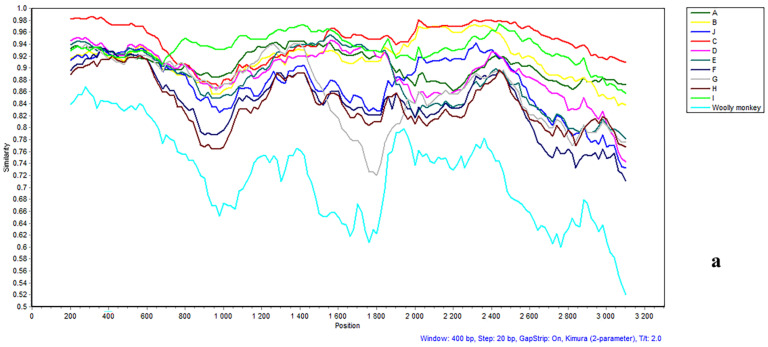

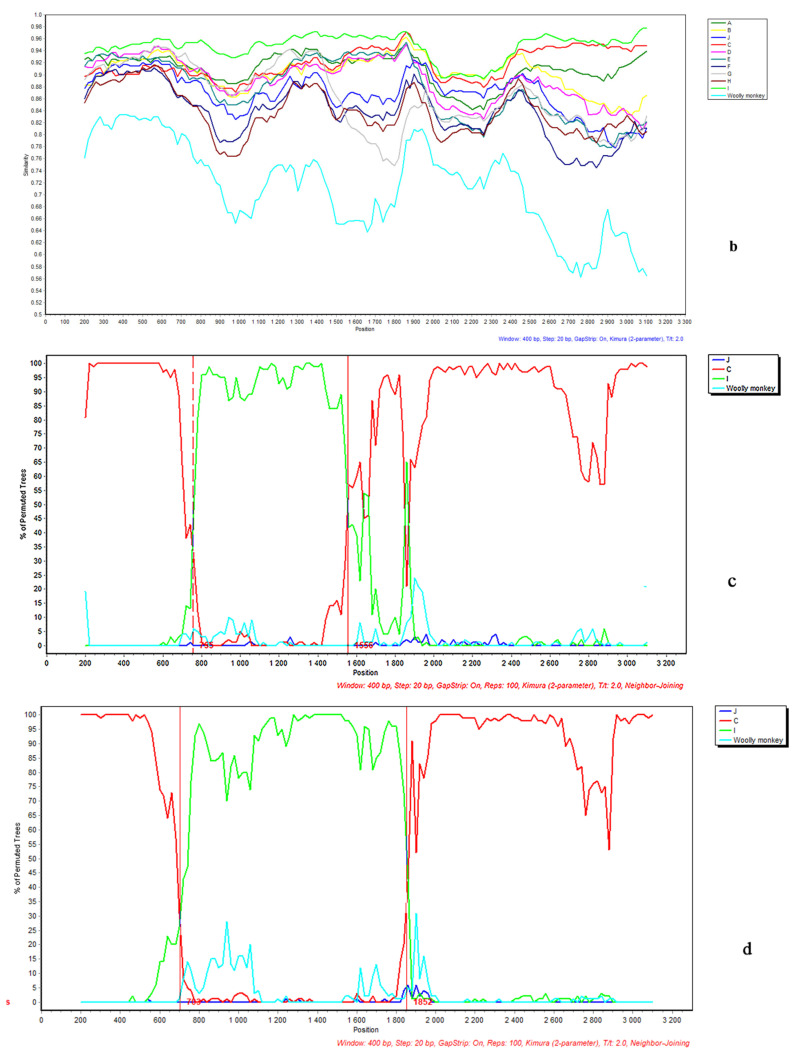
Simplot analysis of the recombination of strains SS584 (group 1 and group 2) (**a**,**b**) shows similarity for each position. (**c**,**d**) shows the percentage of permuted trees (BootScan). P, C, S, and X indicate the polymerase, core, surface, and X genes, respectively.

Evidence of recombination was detected in strain group 1 (SS584-CBS-13 and SS584-CBS-24), group 2 (SS584-CBS-21and SS584-CBS-31) using Simplot 3.5.1 software (Figure 5a,b). The results showed that SS584-CBS-13 and SS584-CBS-24 had no obvious recombination, but SS584-CBS-21 and SS584-CBS-31 had obvious recombination.

The bootscanning result of strain SS584-CBS-21 showed that parts of the genome (nt 1 to 765 and nt 1556 to 3057) were more similar to genotype C, while the remaining (nt 765 to 1556) was more similar to genotype I (Figure 5c). Parts of the genome of strain SS584-CBS-31 (nt 1 to 703 and nt 1852 to 3158) were more similar to genotype C, while the remaining (nt 703 to 1852) was more similar to subgenotype I (Figure 5d). Phylogenetic analyses of both the whole-genome and the S gene sequences further confirmed this. The isolates clustered within genotype C in the S gene tree but belonged to genotype I in the whole-genome tree, with bootstrap support values exceeding 70% in both cases. Clearly, both strains are recombinants between genotype C and genotype I.

## 4. Discussion

To the best of our knowledge, this study is the first designed to compare the accuracy of genotyping based on complete HBV genome sequences determined by NGS and CBS. The principal findings are that genotyping results with sequences obtained from NGS may be inconsistent with those from CBS. CBS can find more subgenotypes and mutations than NGS. Genotyping based on S ORF sequences obtained by direct sequencing may not be reliable in this region with complex genotypes. The strength of the study is that complete genome sequences were used for genotyping, which may provide more information to compare the advantages of NGS and CBS. The major limitation of this study is that none of the study subjects had two serum samples collected in 2011 and 2020, respectively, which may allow us to compare the evolution of HBV genomes in infected individuals.

The ability to conduct high-resolution analyses of viral quasispecies is a major advantage of next-generation sequencing, stemming from its power to read thousands of distinct molecular sequences. Consequently, this technology has seen broad adoption in diverse research fields, for instance, in virological and cancer-related investigations [33]. However, compared to Sanger sequencing, the ability of NGS to identify genotypes remains unclear. It has been reported that the results of NGS genotyping showed a concordance of 95.2% with INNO-LiPA and 100% with Sanger sequencing. However, the sequences used for genotyping covered only 418 nt of the polymerase gene [34]. Another study showed that NGS based on the complete genome is useful to discriminate mixed genotypes detected with INNO-LiPA. Unfortunately, these results were not confirmed by Sanger sequencing [35]. In this study, we compared NGS genotyping results with those of CBS based on the complete genome. Therefore, our study may provide more information on the ability of NGS genotyping.

Numerous studies have demonstrated that the specific HBV genotype correlates with clinical manifestations and can serve as a genetic indicator to predict the progression of the disease [9]. These genotypic variations are particularly relevant to therapeutic outcomes, as they have been shown to impact responses to interferon-α treatment and the potential for achieving a functional cure via HBsAg loss [36]. Therefore, characterizing the viral genotype, along with other prognostic factors, allows for the formulation of customized treatment approaches and informs surveillance strategies, including hepatocellular carcinoma screening protocols [37]. Therefore, the findings of our study are likely important to clinicians.

For detecting mixed-genotype HBV infections, direct sequencing of PCR products is considered inadequate [13]. Clone-based sequencing (CBS) offers a partial solution, but it is hampered by potential selection artifacts and the small sample of clones, which may not be fully reflective of the viral population’s genetic complexity [38]. In contrast, next-generation sequencing (NGS) provides enormous throughput, enabling deeper sequencing capacity [33]. It has been widely reported that for identifying minor variants and simulating quasispecies, NGS demonstrates superior sensitivity and efficiency when compared to the CBS method [17].

Surprisingly, the data from our study did not align with these expectations. The genotyping outcomes derived from NGS sequences were not congruent with those from CBS; in fact, CBS identified more subgenotypes and mutations. This counterintuitive finding might stem from the larger number of complete viral sequences generated through CBS in our specific workflow compared to NGS. It is important to note that our NGS pipeline did account for diversity by treating any haplotype with a frequency of at least 1% as a unique HBV variant. This discrepancy highlights the need for further studies to clarify the optimal application of these methods.

Our sequencing data reveal a complex landscape of HBV quasispecies, characterized by a dynamic equilibrium between dominant and rare variants. The dominant quasispecies, which constitute the majority of the viral population, are believed to represent the fittest viruses in the current host environment, driving ongoing viral replication and disease progression. However, the clinical significance of the rare quasispecies cannot be overlooked. This reservoir of genetic diversity, while quantitatively minor, serves as a critical archive for pre-existing drug-resistant or immune-escape mutations [39,40].

The presence of low-frequency variants can act as a predictive biomarker for treatment failure. For instance, the pre-existence of NA-resistant mutations within the rare quasispecies pool, even before the initiation of therapy, is a well-documented mechanism leading to subsequent virological breakthrough. These rare variants represent a formidable challenge to the host’s immune control and vaccine efficacy. Under selective pressure from neutralizing antibodies or cytotoxic T lymphocytes, a previously rare immune-escape variant can be rapidly selected, outgrow the former dominant population, and lead to immune evasion and chronicity [41,42,43].

It has been reported that a more cost-effective approach to genotyping is to sequence a single ORF (e.g., the surface ORF) instead of the complete genome [13]. The findings from this kind of analysis may be adequate for classifying the primary HBV genotype but are often unsuitable for subgenotype assignment if genetic exchange has taken place [4]. This is because recombination events can interfere with the proper reconstruction of a phylogenetic tree and lead to an incorrect increase in nucleotide divergence values [44]. The misclassification of strains such as subgenotypes B3, B5, and C11 serves as a clear illustration of this analytical pitfall [45]. In this study, we found that four of the five subjects’ genotypes identified in direct sequencing could not be found by NGS/CBS. One subject has the same genotype found by direct sequencing as by NGS and CBS. Clearly, genotyping based on the S gene ORF may not be reliable in regions with complex genotypes.

The high replication rate of HBV and the lack of proofreading activity of its polymerase result in high genetic heterogeneity [2]. Resistance mutations may occur naturally during long-term infection [46]. A wide variation has been observed in the frequency of naturally occurring resistance mutations among patients who have no prior treatment history, with reported prevalence rates spanning from 0% up to 57% [47]. These mutants sometimes may appear predominant and sometimes as minor quasispecies [35,48].

Inter-variant genetic recombination serves as a pivotal mechanism in viral evolution. The occurrence of recombination events disrupts the coherence and consistency of evolutionary histories among genomic regions, including distinct gene segments [49]. HBV recombinant strains between different genotypes/subgenotypes play critical roles in shaping viral genetic diversity and facilitating transmission among human populations [50]. Multiple recombinant strains, such as C/D, A/E, B/C, B/D, and A/G recombinants, have been identified [51,52]. Our study identified two classes of suspected recombinant strains. Subsequent phylogenetic tree, Simplot, and Bootscan analyses confirmed one class as I/C recombinant strains. Intriguingly, another cluster exhibited no detectable recombination signals but displayed an evolutionary divergence exceeding 4% from both I1 and I2. We provisionally classify it as an aberrant subgenotype of I, pending further validation to determine whether it represents a novel subgenotype of I. The identification of these recombinant and aberrant strains aids clinicians in evaluating clinical outcomes and antiviral treatment responses for patients with specific viral genotype infections, enabling precision therapy. However, further epidemiological characterization of such strains is warranted.

In this study, we found that all the study subjects had pre-existing resistance mutations that occurred as minor quasispecies. It remains crucial to determine how the presence of minor viral populations harboring primary drug resistance mutations might influence the therapeutic response to subsequent antiviral treatment.

## Figures and Tables

**Table 1:** HBV reference sequences of genotypes retrieved from GenBank.

HBV Genotype	Subgenotype	Reference GenBank ID	Country of Origin
A	A1	AB116082	Japan
	A2	EU859908	Belgium
	A3	AB194952	Japan
B	B1	AB602818	Japan
	B2	EU939638	China
		AB981582	Japan
	B3	AB976562	Indonesia
	B4	AB115551	Cambodi
	B5	GQ924645	Malaysia
	B6	KP659253	Canada
	B7	GQ358143	Indonesia
	B8	GQ358146	Indonesia
	B9	GQ358152	Indonesia
	B10	MN689123	China
C	C1	GQ35154	Indonesia
		AB074047	Japan
	C2	AB042285	Japan
		FJ386625	China
	C3	X75665	Sweden
	C4	AB04870	Australia
	C5	JN827414	Thailand
		AB241111	Philippine
	C6	AB493847	Indonesia
	C7	EU670263	Philippines
	C8	AP011106	Indonesia
	C9	AP011108	Indonesia
	C10	AB540583	Indonesia
	C11	AB554020	Indonesia
	C12	AB554025	Indonesia
	C13	AB644281	Indonesia
	C14	AB644284	Indonesia
	C15	AB644286	Indonesia
	C16	AB644287	Indonesia
	C17	MG826140	China
D	D1	AB188244	Japan
E	E	AB032431	Liberia
F	F	DQ823094	Argentin
G	G	AF405706	Germany
H	H	AY090460	USA
I	I1	AB231908	Vietnam
		FJ023659	Laos
		FR714490	China
	I2	FJ023664	Laos
		FJ023670	Laos
Woolly monkey	Woolly monkey	AY226578	

**Table 2:** Serological test results of study samples.

Code	Sex	Age	Year	HBsAg	Anti-HBs	HBeAg	Anti-HBe	Anti-HBc	ALT	HBV DNA
SS078	Female	50	2020	+	−	−	+	+	ND *	4.06 × 10^2^
SS078	Female	41	2011	+	−	−	+	+	18.8	ND
SS255	Female	63	2020	+	−	−	+	+	ND	4.59 × 10^2^
SS255	Female	54	2011	+	−	−	+	+	23.8	ND
SS584	male	66	2020	+	+	−	−	+	ND	88.00
SS584	male	57	2011	+	−	−	+	+	21.60	ND
BY640	Female	40	2020	+	−	−	+	+	ND	4.63 × 10^2^
BY640	Female	31	2011	+	−	−	+	+	27.1	ND
CW512	male	52	2020	+	−	−	−	+	ND	88.00
CW512	male	43	2011	+	−	−	−	+	36.5	ND

* ND = No detection.

**Table 3:** Comparison of S genome-based and full-length genome genotyping methods.

Code	Year	NGS-Genotyping		CBS-Genotyping		Sanger-Genotyping (a)	Sanger-Genotyping (b)
		Whole Genome	S Gene	Whole Genome	S Gene	S Gene	S Gene
SS078	2011	ND	ND	ND	ND	B2	O
SS078	2020	C5	C5	C5 and C1	C5 and C1	ND	ND
SS255	2011	ND	ND	ND	ND	B2	O
SS255	2020	B2	B2	B2	B2	ND	ND
BY640	2011	ND	ND	ND	ND	G	I1
BY640	2020	C1	C1	C1	C1	ND	ND
CW512	2011	B2	B2	B2	B2	O	O
CW512	2020	ND	ND	ND	ND	ND	ND
SS584	2011	C1	C1	C1, recombinant, aberrant	C1, aberrant	C2	B
SS584	2020	ND	ND	ND	ND	ND	ND

ND = No detection, O = cannot be genotyped, a: Geno2pheno hbv, b: phylogenetic tree.

**Table 4:** Summary of mutation detection results of NGS and CBS.

Region/Mode	Mutation	Code									
		SS078		SS255		SS584		BY640		CW512	
		NGS	CBS	NGS	CBS	NGS	CBS	NGS	CBS	NGS	CBS
S	T131N	0.00%	0.00%	0.00%	0.00%	0.00%	11.11%	0.00%	0.00%	0.00%	0.00%
	G145K	0.00%	0.00%	0.00%	0.00%	0.00%	11.11%	0.00%	0.00%	0.00%	0.00%
	G145R	0.00%	32.35%	0.00%	0.00%	0.00%	11.11%	0.00%	0.00%	0.00%	0.00%
RT	M250I	0.00%	2.94%	0.00%	0.00%	0.00%	0.00%	0.00%	0.00%	0.00%	0.00%
	V214A	0.00%	0.00%	0.00%	0.00%	0.00%	0.00%	100.00%	97.30%	0.00%	0.00%
C	L100I	0.00%	8.82%	0.00%	0.00%	0.00%	11.11%	0.00%	0.00%	0.00%	0.00%
	L100P	0.00%	0.00%	0.00%	0.00%	0.00%	0.00%	0.00%	2.70%	0.00%	0.00%
	P130T	0.00%	82.35%	0.00%	0.00%	100.00%	88.89%	0.00%	0.00%	0.00%	3.70%
	P135Q	0.00%	0.00%	100.00%	100.00%	0.00%	0.00%	0.00%	0.00%	0.00%	0.00%
	P135A	0.00%	0.00%	0.00%	0.00%	0.00%	0.00%	0.00%	0.00%	7.41%	11.11%
X	C1653T	85.71%	8.82%	0.00%	0.00%	0.00%	0.00%	0.00%	0.00%	0.00%	0.00%
	T1674C	0.00%	2.94%	0.00%	0.00%	0.00%	0.00%	0.00%	0.00%	0.00%	0.00%
PreC	G1896A	100.00%	6.10%	100.00%	100.00%	0.00%	0.00%	100.00%	97.30%	100.00%	100.00%
	G1899A	100.00%	6.10%	0.00%	0.00%	0.00%	0.00%	0.00%	0.00%	0.00%	0.00%
	G1862T	14.00%	0.00%	0.00%	0.00%	0.00%	0.00%	0.00%	0.00%	0.00%	0.00%
BCP	C1673T	0.00%	0.00%	100.00%	100.00%	0.00%	0.00%	0.00%	0.00%	100.00%	100.00%
	T1753C	0.00%	0.00%	0.00%	0.00%	0.00%	0.00%	100.00%	97.30%	0.00%	0.00%
	A1762T	0.00%	96.70%	100.00%	100.00%	8.00%	33.33%	100.00%	100.00%	0.00%	0.00%
	G1764A	0.00%	96.70%	100.00%	96.60%	8.00%	27.78%	100.00%	100.00%	0.00%	0.00%

## Data Availability

All data generated or analyzed during this study are included in this article. Further enquiries can be directed to the corresponding author.

## Abbreviations

The following abbreviations are used in this manuscript:

NGS	Next-Generation Sequencing
CBS	Clone-Based Sequencing
HBV	Hepatitis B Virus
ORF	Open Reading Frame
HIV-1	Human Immunodeficiency Virus Type 1
HCV	Hepatitis C Virus
EIA	Enzyme Immunoassays
ALT	Alanine Aminotransferase
PCR	Polymerase Chain Reaction
RFLP	Restriction Fragment Length Polymorphism
RFMP	Restriction Fragment Mass Polymorphism
MS	Mass Spectrometry
INNO-LiPA	INNO-LIPA (A commercial line probe assay)
HBsAg	Hepatitis B Surface Antigen
HBeAg	Hepatitis B e Antigen
anti-HBe	Antibody to Hepatitis B e Antigen
HCC	Hepatocellular Carcinoma
DNA	Deoxyribonucleic Acid
nt	Nucleotide(s)
BCP	Basal Core Promoter
RT	Reverse Transcriptase
PreC	Pre-Core
PreS	Pre-Surface
S	Surface
P	Polymerase
C	Core
